# Electrospun bioactive polymer biomaterials enriched with collagen and platelet-rich plasma as a platform for *in vitro* chondrogenic differentiation of human mesenchymal stem cells

**DOI:** 10.3389/fbioe.2025.1629912

**Published:** 2025-12-03

**Authors:** Paulina Trzaskowska, Ewa Rybak, Kamil Kopeć, Tomasz Ciach, Piotr Wieciński, Wojciech Święszkowski, Ewa Kijeńska-Gawrońska

**Affiliations:** 1 Centre for Advanced Materials and Technologies CEZAMAT, Warsaw University of Technology, Warsaw, Poland; 2 Faculty of Chemical and Process Engineering, Warsaw University of Technology, Warsaw, Poland; 3 Faculty of Chemistry, Warsaw University of Technology, Warsaw, Poland; 4 Faculty of Materials Science and Engineering, Warsaw University of Technology, Warsaw, Poland

**Keywords:** electrospinning, cartilage tissue engineering, mesenchymal stem cells, chondrogenic differentiation, platelet-rich plasma

## Abstract

**Introduction:**

Electrospun bioactive polymer biomaterials have gained increasing interest as platforms for cartilage tissue engineering due to their ability to mimic the extracellular matrix (ECM) and provide structural and biochemical support for mesenchymal stem cell (MSC) differentiation. The present study aimed to assess the influence of these bioactive compounds on the chondrogenic differentiation of MSCs.

**Methods:**

Poly(L-lactic acid) (PLA) fibrous mats were fabricated using electrospinning techniques, including standard and coaxial electrospinning, to incorporate bioactive components, namely collagen I and platelet-rich plasma (PRP). The wettability, the fibers diameter, degradation of the mats and PRP release profile were assessed. MSC differentiation culture was performed to determine the effect of the mats on the chondrogenic lineage.

**Results:**

The fabricated fibrous mats exhibited distinct morphological and physicochemical characteristics, with core-shell (CS) fibers demonstrating reduced diameters compared to pure PLA and PLA-collagen (Col) fibers. Wettability studies revealed that PRP encapsulation within the PLA shell did not alter the hydrophobic nature of the material, while the presence of collagen significantly enhanced its hydrophilicity. The PRP release profile from CS fibers exhibited a controlled release within the initial 3 days, followed by stabilization. Furthermore, MSC differentiation studies confirmed that both PRP and collagen-enriched fibrous mats supported chondrogenic differentiation over 14-day period, with Col mats demonstrating the highest glycosaminoglycan (GAG) production. The presence of aggrecan, a key chondrogenic marker, was most pronounced on collagen mats and comparable or lower on PRP (CS) compared with PLA, particularly at 14 days.

**Discussion:**

Furthermore, the observations revealed the presence of two critical markers of cartilage differentiation: namely, actin cytoskeletal reorganization and depolymerization. The presented findings highlight the potential of bioactive PLA fibrous mats enriched with PRP and collagen I as promising platforms for cartilage tissue regeneration. The combination of electrospinning techniques enables tailored fiber structures that support chondrogenesis, offering a potential alternative for tissue engineering applications.

## 1 Introduction

Cartilage is a distinctive form of connective tissue that possesses a smooth, elastic fibrous structure providing load-bearing function and enabling frictionless joint movements. It envelopes and protects the ends of long bones at the joints. Moreover, cartilage constitutes a structural component of the rib cage, the ear, the nose, the bronchial tubes, the intervertebral discs, and many other body components. The extracellular matrix (ECM) of cartilage is very dense, mainly composed of water, collagen, proteoglycans, and glycosaminoglycans, with chondrocyte cells embedded within. The specific structure of this tissue lacks blood vessels, lymphatics, and nerves, which in turn causes significant limitations in its intrinsic regeneration capability after injury or damage (Wang et al., 2024). Conventional treatment strategies, including microfracture, autologous chondrocyte implantation, and osteochondral grafting, often result in the formation of fibrocartilage, which lacks the mechanical flexibility of native cartilage (Zhao et al., 2021). To address these limitations, biomaterials-based regenerative approaches have gained significant attention. Among these, electrospinning has emerged as a powerful fabrication technique for developing nanofibrous scaffolds that mimic the native ECM, and electrospun bioactive polymer biomaterials (EBPB) have emerged as a promising strategy for cartilage tissue repair and regeneration (Yilmaz and Zeugolis, 2020). Electrospinning is a unique technique that employs an electric field to generate nanofibers from diverse materials, including natural and synthetic polymers, and composites (Alves da Silva et al., 2010). Electrospun structures provide a high surface area, tunable porosity, and mechanical properties analogous to natural tissues. The resulting fibers can be fabricated into various constructs, including tissue-engineered scaffolds that mimic the cartilage extracellular matrix, providing a suitable environment for cell habitation and homeostasis (Wu and Hong, 2016). These scaffolds can be modified to align with specific requirements for tissue repair, like optimal mechanical strength, biocompatibility, and biodegradability (Wasyłeczko et al., 2020). The utilization of EBPB presents several advantages over traditional methodologies, rendering it an appealing proposition for cartilage tissue engineering. One of the principal advantages of utilizing EBPB for cartilage tissue repair is its capacity to emulate the structure and functionality of natural cartilage (Zulkifli et al., 2023). The nanofibrous structure provides a high surface area for cell attachment and proliferation, while also facilitating the diffusion of nutrients and waste products (Ding et al., 2021). Additionally, the mechanical properties of the scaffolds can be modified to match those of natural cartilage, ensuring adequate support for tissue regeneration (Wu and Hong, 2016). Moreover, EBPB has been demonstrated to facilitate chondrogenic differentiation of mesenchymal stem cells, resulting in the development of mature and functional cartilage tissue (Sorrell et al., 2018).

Various types of synthetic polymers have been successfully proposed as matrices for cartilage engineering, such as polylactic acid (PLA), polyglycolic acid (PGA), and polycaprolactone (PCL), as well as natural materials like collagen type I or alginate. PLA is a biodegradable and biocompatible polyester that is used in biomedical applications and has been approved by the FDA. PLA is now widely used in surgery, orthopaedics, orthodontics, traumatology, and other branches of medicine (Herrero et al., 2021). Its efficacy in tissue engineering as a scaffold for the growth and differentiation of mesenchymal stem cells to obtain different cell lineages has also been investigated (Wayne et al., 2005). The current research on EBPB for cartilage tissue repair is focused on the improvement of the mechanical and biological properties of the fabricated scaffolds. Recent advances in three-dimensional electrospinning have facilitated the fabrication of constructs with tailored geometries, enabling their adaptation to the dimensions and configuration of the damaged site (Flores-Rojas et al., 2023). Furthermore, there are ongoing research studies on how to incorporate growth factors and other bioactive molecules into the scaffolds to enhance the regenerative potential of the tissue (Alves da Silva et al., 2010). Despite the current limitations of EBPB in cartilage tissue repair, including the necessity for further optimization of the scaffold design and fabrication techniques, the potential for this approach to revolutionize cartilage tissue engineering is significant (Zhou et al., 2018), and advanced electrospinning strategies, such as core-shell and blended polymer systems, allow for the incorporation of active agents within the fibrous structures (Kijeńska-Gawrońska et al., 2019).

As documented, the integration of bioactive molecules such as collagen and platelet-rich plasma (PRP) into EBPB promotes chondrogenic differentiation, thereby substantiating the potential of this approach for tissue engineering applications. Both collagen and PRP are essential components in the enhancement of adhesion, proliferation, and chondrogenic differentiation of MSC (Diaz-Gomez et al., 2014; Mardani et al., 2013; Le et al., 2020; Jiang et al., 2019). Collagen, a principal component of the extracellular matrix (ECM) in cartilage tissue, provides vital structural support to chondrocytes. Meanwhile, PRP contains growth factors and cytokines that have been demonstrated to stimulate cell proliferation and differentiation (Mardani et al., 2013; Le et al., 2020). The incorporation of collagen and PRP into EBPB has a synergistic effect that supports the chondrogenic differentiation of MSC, thereby enhancing the overall effectiveness of the designed scaffolds (Teixeira et al., 2020). Also, it has been reported that the addition of both components to a polymeric scaffold improves scaffold bioactivity and mechanical properties, enhancing chondrogenic differentiation even without specific induction media (Jiang et al., 2018; Wu et al., 2020). Overall, the use of EBPB materials with collagen and PRP represents a promising approach for promoting chondrogenic differentiation of MSCs *in vitro*, with significant potential for application in tissue engineering and regenerative medicine (Zhao et al., 2021; Flores-Rojas et al., 2023; Nicolas et al., 2020; Li et al., 2021; Chen, 2022).

The present study subjected MSCs to chondrogenic differentiation culture on electrospun poly-l-lactic acid (PLA) mats of the following types: pure monolithic polymeric fibrous mats lacking any biological factors, core-shell mats encapsulated with platelet-rich plasma (PRP), and blended mats containing collagen I. The objective was to assess the influence of bioactive agents incorporated in PLA mats on human MSCs undergoing chondrogenic differentiation during *in vitro* culture. Produced fibrous scaffolds of all types were characterized in terms of their morphology, structure, physico-chemical and mechanical properties, and degradability. Moreover, the PRP proteins’ release profile from core-shell mats was assessed, and then all materials were subjected to MSC culture. The culture was carried out for 3, 7, and 14 days. DNA content of MSCs contacting the materials was determined, and chondrogenic differentiation of MSCs was evaluated with quantitative GAG content analysis and quantitative and qualitative aggrecan fluorescence detection. It was hypothesized that the incorporation of growth factors and proteins like PRP or collagen I could facilitate the chondrogenic differentiation process. Consequently, these materials could potentially serve as bioactive implants for the stabilization and regeneration of cartilage tissue, for instance, in the context of intervertebral disc trauma.

## 2 Materials and methods

### 2.1 Preparation of fibrous mats

#### 2.1.1 Solutions preparation

**FIGURE 1:**
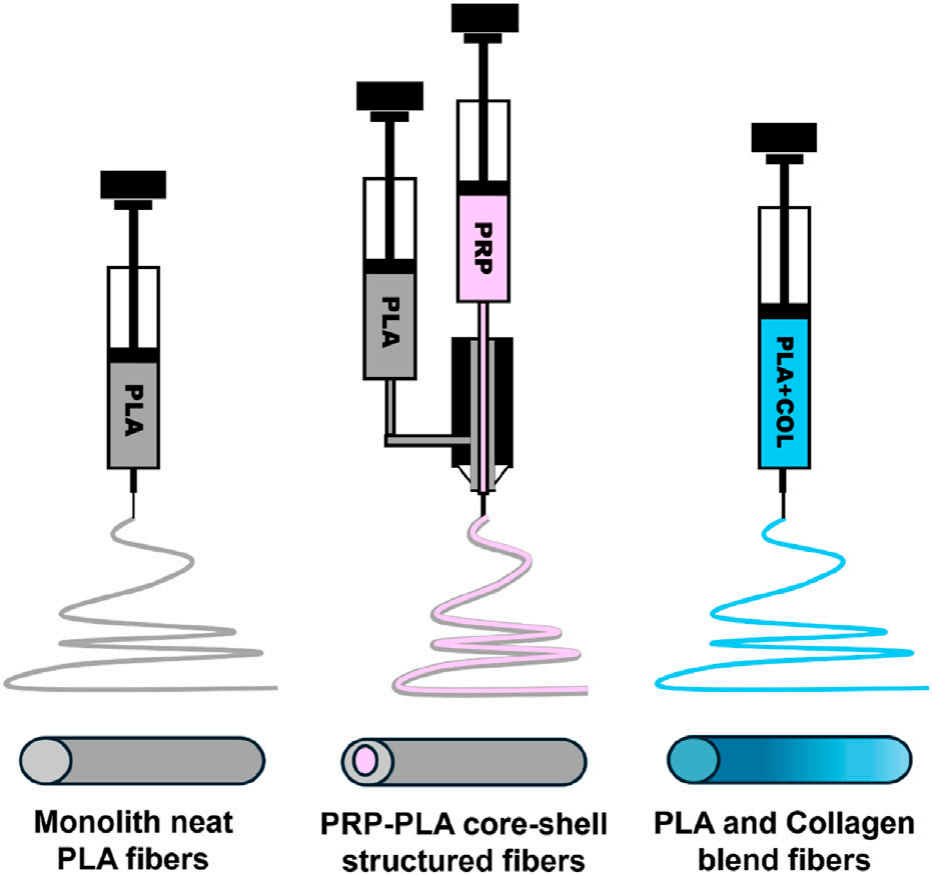
Schematic showing the type of electrospinning probes and the structure of the obtained fibers.

Three types of fibrous scaffolds have been prepared in this study: monolith pure polymeric PLA [PLA], blended PLA-collagen [Col], and PRP-PLA core-shells [CS] (Figure 1).

For the preparation of the solutions for electrospinning of monolith PLA fibers and PLA shell of core-shell fibers, PLA powder (PURASORB PL18, Corbion, Netherlands) was dissolved in 1,1,1,3,3,3-hexafluoro-2-propanol (HFP) [FL3409, Th.Geyer Sp. z o.o., Poland] and mixed overnight to form 13% (w/v) and 14% (w/v) mixtures, respectively. In the case of PLA-collagen blends, PLA and collagen (Atelocollagen CLP-01, KOKEN, Japan) in a ratio of 70:30 were dissolved in HFP and mixed overnight to form a 13% (w/v) solution. The core solution phase was composed of platelet-rich plasma (PRP).

PRP was obtained in accordance with the following protocol. Buffy coats were obtained from 40 healthy male donors (20–30 years old; blood groups O, A, B, and AB) provided by the Regional Centre for Blood Donation in Warsaw. All donors were pre-screened for HBsAg, anti-HCV, HCV RNA, anti-HIV-1/2, and HIV RNA to ensure biosafety and consistency. After isolating PBMCs using Ficoll-Paque density gradient centrifugation (Baran et al., 2025), the remaining PRP supernatant was collected without disturbing the Ficoll interface and stored at −80 °C until use.

#### 2.1.2 Electrospinning of PLA and Col fibrous mats

Both types of fibers were electrospun using similar process parameters and conditions. After mixing, prepared solutions were transferred separately to 5 mL plastic syringes attached to 27 G blunted stainless steel needles connected to the high voltage of 8 kV (Gamma High Voltage Research, United States). The feed rate of solutions was fixed at 0.8 mL/h, and the distance between the needle electrode and the aluminum foil grounded collector was set at 12 cm.

#### 2.1.3 Electrospinning of CS fibrous mats

The PRP core and PLA shell solutions were individually placed into 3 mL and 5 mL syringes and attached to the coaxial needle in the needle system consisting of an internal 25 G core needle and an outer 18 G shell needle. The flow rates of PRP and PLA solutions were 0.05 mL/h and 1.0 mL/h, respectively. A high voltage of 17 kV was applied to the system through the outer needle, and the distance between the needle system and the aluminum foil collector was set at 14.5 cm.

All the fibrous materials, after electrospinning, were transferred to a vacuum drier (Vacucell, MMM Medcenter Einrichtungen GmbH, Germany) and dried for at least 24 h under 0.05 bar.

### 2.2 Morphological characterization of the fibrous mats

The morphology of fabricated PLA, CS, and Col fibrous mats was investigated using a scanning electron microscope (Phenom, FEIQuanta, Netherlands) at an accelerating voltage of 10 kV after sputter coating all samples with a double 7 nm gold layer using a sputter coater (Leica EM SCD500, Leica, Germany). The average diameters were evaluated for particular materials by calculating diameters from 100 randomly selected points on SEM images using image analysis software (ImageJ, National Institute of Health, United States).

A cross-section and subsequent morphology observation of CS fibers were performed using the Thermo Scientific Helios 5 PFIB CXe field emission scanning electron microscope (FE-SEM), equipped with a xenon (Xe) plasma-focused ion beam (FIB) column. A thin gold (Au) coating was initially deposited on the samples to enhance surface conductivity and minimize charging effects. Before initiating cross-sectioning, the region of interest was protected with an additional tungsten (W) layer to prevent surface damage during milling. The cross-section was created using an ion beam with an acceleration voltage of 30 kV and a beam current of 1 nA. Morphological characteristics of the material were then observed using the secondary electron (SE) signal.

### 2.3 Characterization of the physicochemical and mechanical properties of the mats

The wettability of the as-spun fibrous mats was evaluated by investigation of static water contact angle (WCA) values using a goniometer (OCA 20, Dataphysics, Germany). During the tests, a 0.5 µL droplet of water was gently placed on the surface of the mats placed on the testing table, and as soon as the droplet touched the sample, an image was taken, and WCA was calculated using dedicated SCA 20 software. At least five measurements were taken for each sample.

Fourier-Transform Infrared Spectroscopy (FTIR) of all fabricated scaffolds was performed to investigate functional groups and chemical bonds present on the surface of the biomaterials. Measurements were carried out using the attenuated total reflectance (ATR) mode. Each sample was scanned 256 times at a resolution of 4 cm^−1^ over the frequency range of 4000–400 cm^−1^ using a Fourier transform infrared spectrophotometer (Thermo Fisher Scientific model Nicolet iS50).

The tensile properties of the fibrous mats were investigated using a tabletop tensile tester (Instron 5943, Instron, United States) at a crosshead speed of 5 mm/min. All tested samples were prepared in rectangular shapes measuring 40 × 5 mm and secured in hydraulic clamps during the tests.

### 2.4 Quantitative analysis of total PRP protein amount released from PLA core-shell fibers

In order to examine the release of total PRP protein, 10 mm diameter discs were cut out from CS fibrous biomaterials containing PRP and sterilized by UV irradiation for 30 min on each side. The discs were transferred to a 24-well plate, and 1 mL of sterile PBS with a pH of 7.4 (Sigma Aldrich, Germany) was added to each sample. Further, the mats were incubated at 37 °C in the dark (mixing was not applied). At selected time points, 200 µL of the solutions were collected and replaced with 200 µL of fresh PBS. The collected samples were stored at −18 °C. The determination of total protein concentration in the collected samples was performed using the QuantiPro™ BCA Assay Kit (Sigma-Aldrich, Germany). Analyses were performed following the manufacturer’s instructions. To calculate the total protein concentration, a standard curve was prepared for bovine serum albumin (BSA) solutions (the BSA standard solution was included in the QuantiPro™ Kit). Six discs were examined for each time point (n = 6). The results were presented as the cumulative protein release profile–the mass of the PRP protein released per the area of the PLA mats.

Additionally, to examine PRP release kinetics from fibrous constructs, zero-order, first-order, Korsmeyer–Peppas, Higuchi, and Hixson–Crowell mathematical models were implemented (Cam et al., 2021). The regression coefficients (*R* ^2^) for each model are given in Supplementary Figure S1, and the highest (*R* ^2^ = 0.950) was found for zero-order kinetics (Supplementary Figure S1b).

### 2.5 Evaluation of the degradability of the fibrous mats

The capacity of hydrolytic degradation of fabricated PLA, CS, and Col fibrous scaffolds was evaluated by soaking a sample portion of each type of mat in PBS (pH 7.4) for 14 days. After 1, 3, 5, 7, 10, and 14 days, three samples of each kind were removed, washed carefully with DI water, and dried under vacuum (0.5 bar, 25 °C) for at least 48 h. Further, the mass of the samples was determined, and the percentage mass loss was calculated for each kind of scaffold (Kijeńska-Gawrońska et al., 2019). Moreover, the changes within the morphology of the tested materials were evaluated after 3, 7, and 14 days of incubation in PBS using SEM.

### 2.6 Cytotoxicity evaluation

The cytotoxicity evaluation has been performed in accordance with the ISO 10993-5 and 10993-12 standards (International Organization for Standardization, 2009; International Organization for Standardization, 2012). First, L929 murine fibroblasts were cultured for 3 days to reach confluence in RPMI 1640 medium supplemented with 10% heat-inactivated FBS and 1% PS in 5% CO_2_ incubator at 37 °C. Next, cells were detached with 0.05% trypsin-EDTA, seeded in 96-well plates at 10^4^ per well in 100 µL of supplemented medium, and cultured for 24 h. At the same time, to obtain extracts, samples were sterilized under UV for 30 min on each side, washed thrice in PBS, and placed in supplemented medium, keeping the surface area exposure at 6 cm^2^/mL, and incubated on a shaker at 37 °C in a humidified CO_2_ incubator for 72 h. An extraction ratio of 6 cm^2^/mL is a standard parameter in ISO 10993-12, which outlines sample preparation for ISO 10993-5 cytotoxicity tests, with this specific ratio typically applied to medical devices thinner than 0.5 mm to extract potentially harmful substances under realistic conditions. After discarding the medium from wells with cells, 100 µL of each solution was added to the wells, and the cells were incubated for another 24 h. Further, the release medium was removed, replaced with 100 μL of fresh RPMI 1640 and 10 μL of CCK-8 solution, and incubated for 2 h at 37 °C. The absorbance of the obtained dyes was measured at 450 nm in a fresh 96-well plate using an Infinite M200 reader (Tecan, United States). The viability of the cells was quantified by comparing it to cells grown in control medium and presented as a percentage of the control. All samples were tested in triplicate in three technical and two biological repetitions.

### 2.7 Differentiation culture of MSCs on fibrous mats

MSCs (ThermoFisher, A15652) were expanded according to the manufacturer’s instructions (MesenPro medium, ThermoFisher, 12746012), supplemented as recommended with GlutaMAX 35050061; culture conditions: 21% O_2_, 5% CO_2_, 37 °C. Just before differentiation, all scaffolds (flat discs, 10 mm) and control materials (glass for cell culture, 10 mm) were sterilized [UV irradiation for 30 min on both sides in 48-well plates, then immersed in DMEM with 1% penicillin-streptomycin (pen-strep, 1% v/v, ThermoFisher, 15140122)] and placed overnight in cell culture incubator at 37 °C, 5% CO_2_ using inserts to prevent the materials floating. All material variants subjected to differentiation culture are described in Table 1, with a description of the medium composition used in cell culture.

**TABLE 1:** Description of materials variants used for MSC culture and culture media used for each variant.

Biomaterial variant	Description	Medium description with final concentration of ingredients
PLA	Electrospun pure PLA monolithic fibrous mats without additional ingredients or modifications	Differentiation medium - DMEM with Phenol Red, with 4.5 g/L glucose - ITS + Premix Tissue Culture Supplement – 1% - dexamethazone - 100 nM - ascorbate-2-phosphate, 50 ug/mL - proline, 40 ug/mL - penicillin-streptomycin 100x, 1% - human tissue growth factor TGF-β3 10 ng/mL
CS	Electrospun PRP-PLA fibrous mats, core-shell type of fibers, containing PRP.	Differentiation medium, as above
Col	Electrospun PLA-collagen blends fibrous mats, containing type I collagen	Differentiation medium, as above
C	Control material – glass slides sterilized with 70% ethanol and rinsed with sterile culture medium	Non-differentiation medium - DMEM with Phenol Red, 1 g/L glucose - FBS, 10% - penicillin-streptomycin 100x, 1%

The sterilized fibrous discs of all types and controls were placed in a sterile 48-well plate. MSCs (passage 3) were seeded on the materials at a density of 1*10^5^ cells/100 µL using the technique of micromass culture (Challapalli et al., 2022). Briefly, the cell suspension was diluted in DMEM supplemented with FBS (10% v/v, ThermoFisher, 10500064) and pen-strep and then added at 100 µL per disc. After 3 h culture in normoxia (21% O_2_, 5% CO_2_, 37 °C), 1 mL of supplemented differentiation medium was added [DMEM supplemented as described in Table 1: with universal culture supplement ITS + Premix Tissue Culture Supplement (Corning, 354352)], dexamethasone (MERCK, D4902-500 MG), ascorbate-2-phosphate (MERCK, A8960-5G), proline (Sigma Aldrich, BioReagent, ≥98.5%), pen-strep and with 10 ng/mL growth factor TGF- β3 (MERCK, SRP3171, 10 µg). DMEM medium supplemented with 10% FBS and 1% pen-strep was added to control wells (glass slides, medium without any differentiating factors). In all wells, the medium was exchanged every 2–3 days. Differentiation culture was carried out for 3, 7, and 14 days. For further analyses, it was assumed that differentiating cells adhered to the material surface and were also present in the medium as floating aggregates.

### 2.8 Analysis of cellular DNA concentration

The CyQUANT™ Cell Proliferation Assay kit (Thermofisher, C7026) was used to determine the DNA content. At given time points: 3, 7, and 14 days, medium with floating cell aggregates was aspirated from above the materials, put in Eppendorf tubes, and centrifuged. The resulting pellets, as well as fibrous mats with adhered cells, were frozen at −70 °C. Before analysis, a standard curve was prepared based on a series of dilutions of the DNA standard.

Before measurement, analysis solutions were prepared according to the instructions of the kit’s manufacturer. Briefly, CyQUANT™ dye was warmed up to room temperature, the cell lysis buffer was diluted in distilled water, and the RNAse stock solution (Thermofisher, R6513) was prepared in the lysis buffer (final activity 1,35 Kunitz units/mL). Frozen samples from 3 time points were thawed. Empty wells were used as blanks. Diluted RNAse solution was added to each well, including blank, and incubated in the dark for 1 h at RT. The CyQUANT™ dye solution was then added to each well and incubated for 5 min in the dark at RT. After that, the absorbance of the samples and the blank was measured at 480 nm. The DNA content in all samples was calculated using the standard curve.

### 2.9 Immunostaining of MSCs cytoskeleton, nuclei, and aggrecan

Fibrous mats with adhered cells were washed in PBS and transferred to a new well plate. For fixation, the materials were incubated in 4% paraformaldehyde in PBS for 15 min at RT, and thoroughly rinsed with PBS (3 × 5 min on a shaker at RT). To permeabilize cell membranes, 0.2% Triton X-100 in PBS was added to the wells and incubated for 4 min on a shaker. The materials were again rinsed very thoroughly with PBS (as above). For blocking, 0.2% BSA in PBS was added to the wells and incubated for 1 h on a shaker. After washing with PBS as above, the mouse primary anti-aggrecan antibody (Abcam, ab3778) diluted 1000-fold in PBS buffer was added, and the well plate was left overnight at 4 °C. The next day, the materials were washed with PBS. The plate was wrapped in aluminum foil and incubated for 1 h with an anti-mouse secondary antibody (Abcam, ab150091) diluted 500-fold in PBS. After this, the materials were rinsed with PBS and incubated for 1 h with AlexaFluor 488 Phalloidin (Thermofisher (Invitrogen) A12379) diluted 400-fold in PBS at RT. Materials were then rinsed thoroughly with PBS, and DAPI solution (ThermoFisher, 62248) diluted 100-fold in PBS was added for 10 min. Then the samples were rinsed with PBS and kept in fresh PBS in the refrigerator until imaging.

Confocal images of immunostained samples were acquired using consistent microscope settings for all groups. For each of the three scaffold types (PLA, CS, Col) and at each of the two time points (3 and 14 days), a minimum of three non-overlapping fields per sample variant were recorded. Image quantification was performed using image analysis software (ImageJ, National Institutes of Health, United States) (Schneider et al., 2012). Images were analysed as follows: nuclei were segmented, and for each cell, the aggrecan signal in the cytoplasmic/perinuclear region was evaluated. Aggrecan-positive cells were defined as those exhibiting specific aggrecan staining. For each image, the percentage of aggrecan-positive cells was calculated relative to the total number of nuclei, and these image-level values were averaged to obtain a single mean per condition. The data is presented as mean ± standard deviation. The differences between the groups were assessed using one-way ANOVA with Tukey’s *post hoc* test. A p-value of ≤0.05 was considered to be significant.

### 2.10 Quantitative analysis of glycosaminoglycans (GAG) content

The Blyscan™ kit (Biocolor, B1000) was used to determine the amount of glycosaminoglycans (GAG) produced. The manufacturer’s instructions were followed. After 3, 7, and 14-day cultures, well plates with fibrous mats with adhered cells, as well as cell aggregates floating in medium, were frozen at −70 °C. Before analysis, sodium phosphate buffer (pH = 6.4) and 50 mL of extraction reagent with 5 mg papain (Merck, P4762) were prepared. On the day of the experiment, the materials were rinsed with PBS, and the samples were incubated with the extraction reagent with papain at 65 °C for 3 h with occasional stirring. The digested extracts were centrifuged (10,000 x g, 10 min), and the supernatant was collected. Blanks, reference standards, and test samples (all in duplicates) were prepared in Eppendorf microcentrifuge tubes. One ml of Blyscan™ staining reagent was added to each tube, the tubes were mixed on a shaker for 30 min, then centrifuged (13,000 x g, 10 min). The resulting navy blue precipitates were collected. Half a milliliter of dissociation reagent was added to each tube, and stirred until the precipitates were completely dissolved. Two hundred microliters of each sample was transferred to individual wells of a 96-well plate. The absorbance of blanks (sterile water), standards, and test samples was measured at 656 nm. The increase in GAG content was calculated using a standard curve.

### 2.11 Statistical analysis

Data are presented as mean ± standard deviation. Group differences were evaluated with a one-way analysis of variance ANOVA and Tukey’s honestly significant difference test was performed, and p ≤ 0.05 was considered statistically significant.

## 3 Results

### 3.1 Morphology of the fibrous mats

**FIGURE 2:**
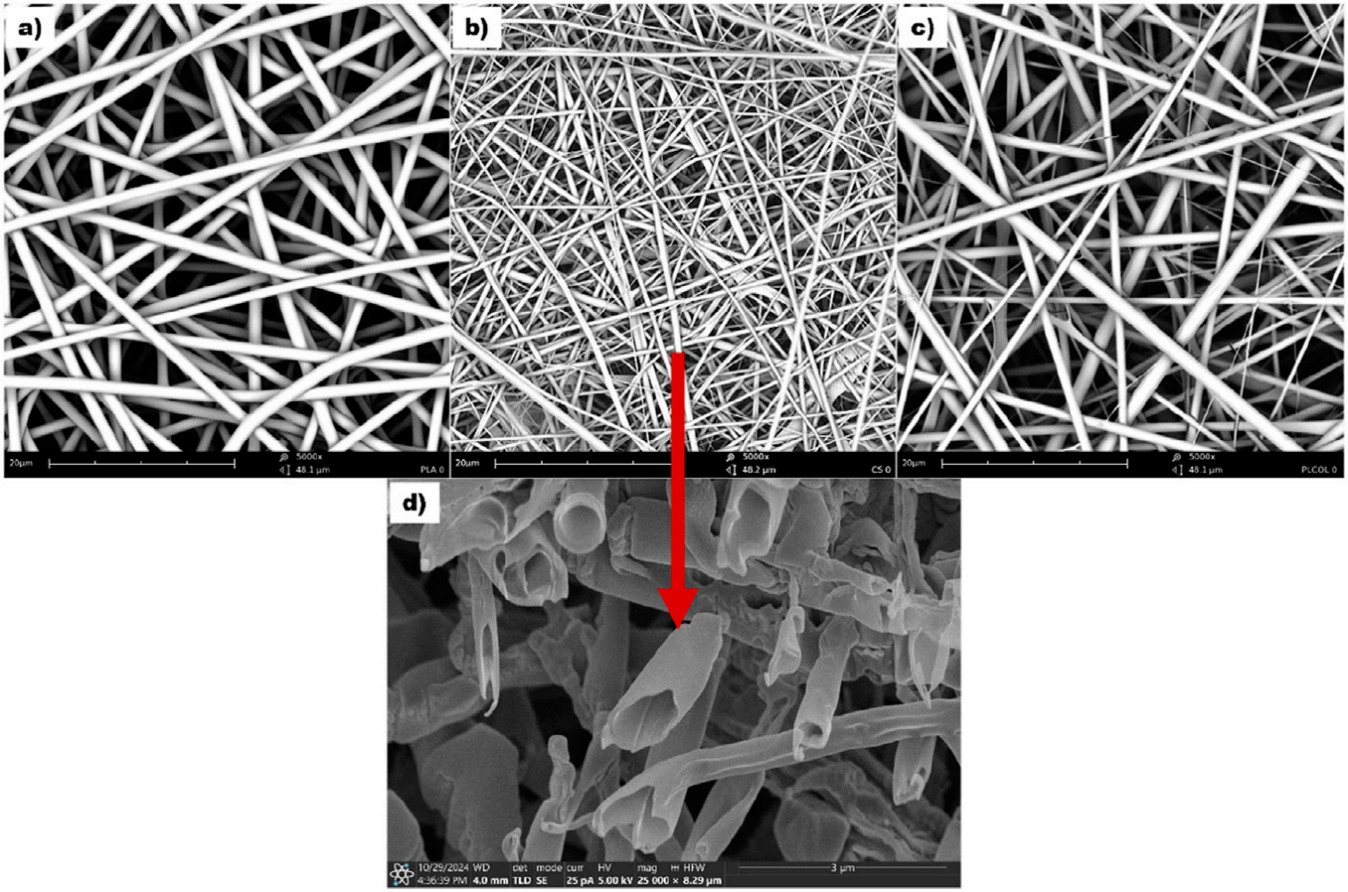
SEM images of **(a)** PLA, **(b)** CS, and **(c)** Col fibrous mats; **(d)** cross-section of CS fibers.

Randomly oriented electrospun fibers of three different compositions were fabricated using standard and coaxial electrospinning methods under optimized conditions. Figure 2 shows the morphology of the obtained PLA, CS, and Col mats, and the cross-sectional image of CS core-shell fibers. The average diameter values of the neat polymeric PLA fibers and blended Col fibers were comparable, measuring 1.43 ± 0.59 µm and 1.50 ± 0.54 µm, respectively. CS fibers were significantly thinner, with an average diameter of 0.73 ± 0.46 µm. The SEM image of the cross-section of the fibers presented in Figure 2d confirmed the core-shell character of CS fibers obtained by coaxial electrospinning.

### 3.2 Physico-chemical and mechanical properties of the fibrous mats

**FIGURE 3:**
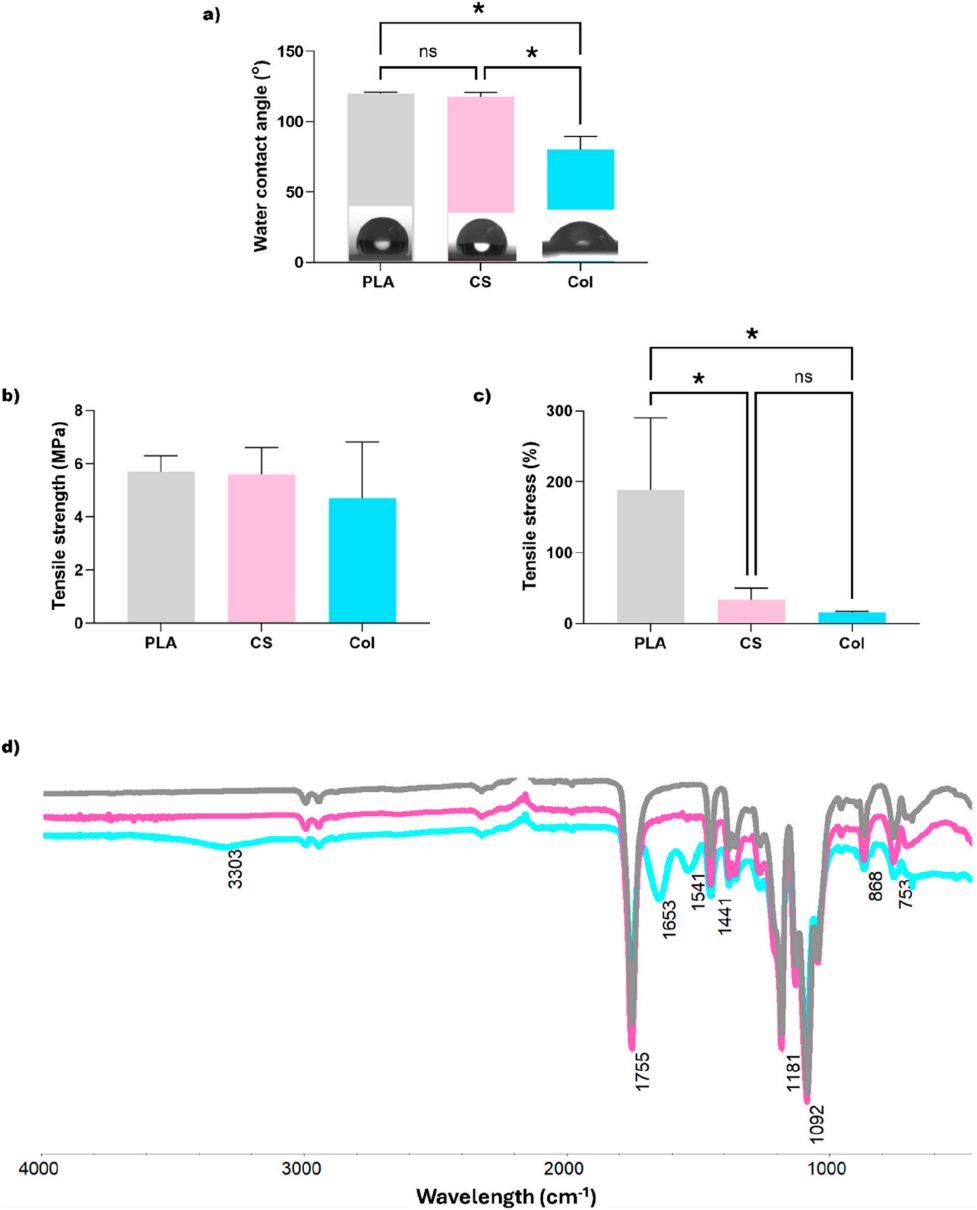
**(a)** Water contact angle measurements, **(b)** tensile strength, **(c)** strain at break values, and **(d)** comparison of ATR-FTIR spectra recorded for investigated electrospun mats: grey–monolith neat PLA, pink–CS, blue–Col. Statistical analysis was performed by one-way analysis of variance (ANOVA) and *post hoc* Tukey’s test, * indicates significance when p ≤ 0.05 (n = 5).

Performed water contact angle measurements (Figure 3a) revealed the hydrophobic nature of the PLA and CS fibrous mats with WCA of 120.12° ± 0.77° and 117.64° ± 3.20°, respectively. On the other hand, Col mats obtained by electrospinning from the blend of PLA and collagen exhibited a hydrophilic nature of their surface with a WCA of 80.04° ± 9.44°.

Figures 3b, c illustrate the obtained tensile strength and tensile stress values for electrospun materials of all types, represented as graphs for comparison. It can be seen that the highest tensile strength of 5.69 ± 0.61 MPa with the highest elongation at break of 188.1% ± 102% was obtained for monolith neat PLA fibers, while the average tensile strength for the fibers enriched with bioactive compounds was 5.60 ± 1.01 MPa with tensile strain of 33% ± 17.1% for CS and 4.68 ± 2.13 MPa with tensile strain of 15.4% ± 1.6% for Col. However, the differences in the tensile strength of all types of fibers were statistically nonsignificant. It can also be observed that the addition of natural components drastically decreased the ductility of the fabricated fibrous materials regardless of their structure.

Investigation of the functional groups and chemical composition of the electrospun fibrous mats was carried out using ATR-FTIR, and a comparison of recorded spectra is presented in Figure 3d. Absorption peak at 1755 cm^−1^ corresponding to C=O bond stretching characteristic for ester groups of PLA can be seen within the spectra of all tested materials, along with asymmetric C-O-C stretching vibrations showing peaks at 1,181 cm^−1^ and 1,092 cm^−1^. With regards to the FTIR spectrum of Col mats, additional characteristic bands associated with the presence of the protein can be observed at 1,653 cm^−1^ and 1,541 cm^−1^ These are related to amide I and amide II, indicating the presence of NH-groups on the surface of these mats (Kijeńska-Gawrońska et al., 2019).

### 3.3 Protein release from PLA core-shell fibers loaded with PRP

**FIGURE 4:**
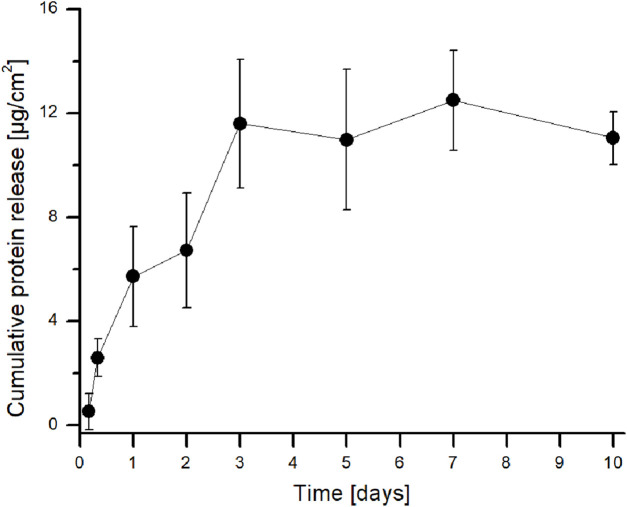
Cumulative protein release from CS fibers loaded with PRP presented as cumulative released protein mass (µg) with regard to fibrous mat area (cm^2^). SDs are presented as thin vertical lines. Six discs were examined for each time point (n = 6).

The cumulative protein release profile is illustrated in Figure 4. The protein content in the PBS reaches a plateau phase after 3 days of release, and from the third day onwards, the protein content remains constant. The results of the 1-h and 4-h incubation periods were below the level of detection.

The release kinetics of PRP from CS fibrous constructs were analyzed using several mathematical models, including zero-order, first-order, Higuchi, Hixson–Crowell, and Korsmeyer–Peppas models, which are presented in Supplementary Figure S1. Among implemented models, the best fitting was observed for the zero-order (*R* ^2^ = 0.950), Higuchi (*R* ^2^ = 0.940), and Korsmeyer–Peppas (*R* ^2^ = 0.945), indicating a consistent and predictable release pattern. In contrast, the first-order (*R* ^2^ = 0.784) and Hixson–Crowell (*R* ^2^ = 0.832) models showed lower correlation coefficients, suggesting that neither degradation nor dissolution was a dominant mechanism influencing PRP release from the fibers (Costa, 2001). Moreover, the strong fit to the zero-order model suggests that PRP was released at a relatively constant rate over time, which is beneficial for maintaining stable therapeutic levels. The good correlation with the Higuchi model indicates that diffusion through the matrix is a major driving force of PRP release. Furthermore, the excellent fit to the Korsmeyer–Peppas model, with a calculated release exponent of n = 1.085, suggests a super case-II transport mechanism, meaning that the release is governed not only by diffusion but also by relaxation or erosion of the matrix, resulting in a controlled and sustained release profile (Korsmeyer et al., 1983).

### 3.4 Degradability of the fibrous mats

**FIGURE 5:**
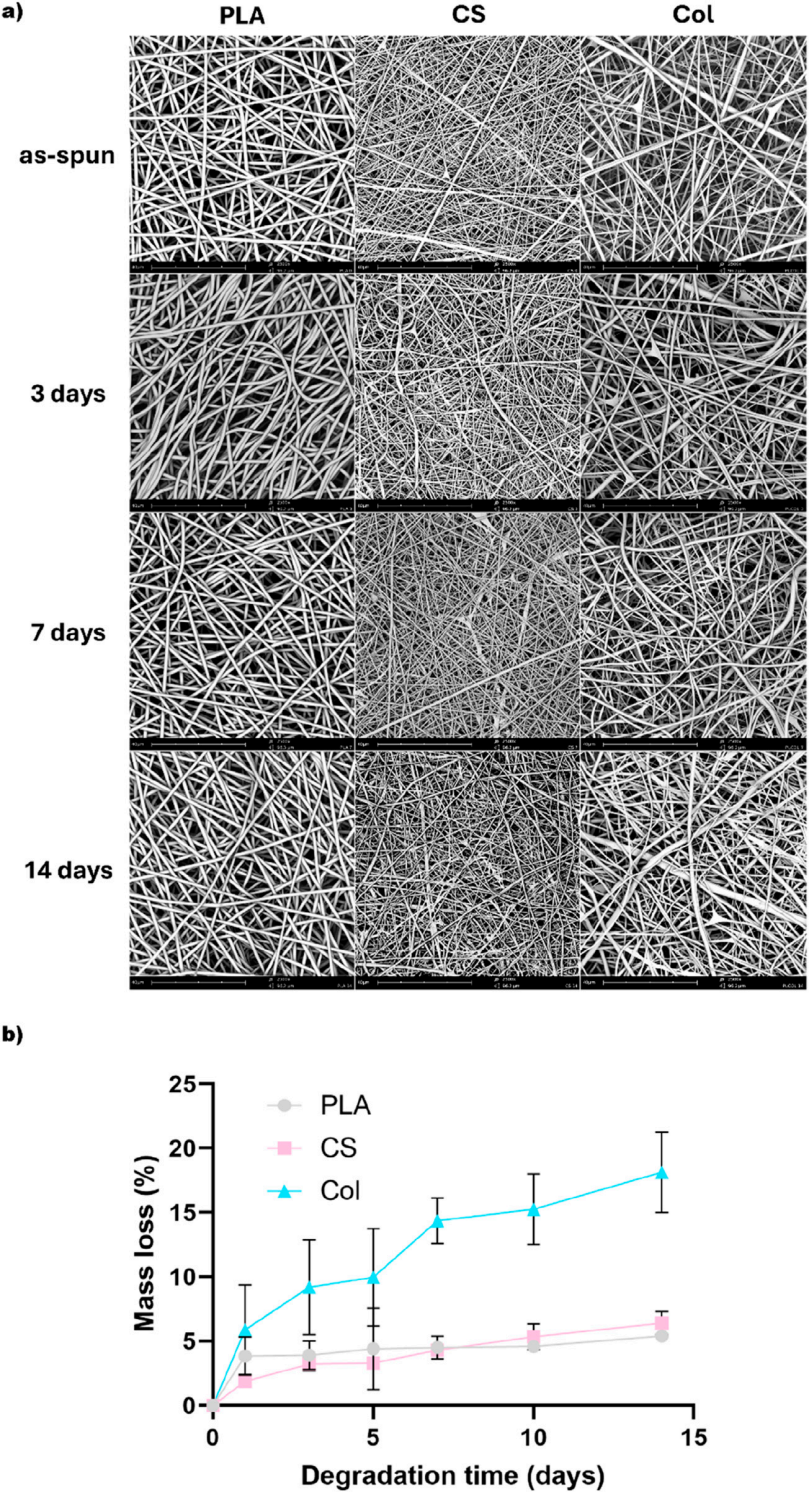
**(a)** morphology of the fibers after fabrication and changes in morphology after 3, 7, and 14 days of degradation; **(b)** graph showing weight loss of the scaffolds during 14 days of degradation in PBS.

Figure 5 presents the results of the degradation study of PLA, CS, and Col fibrous mats *in vitro*. After 14 days of the investigation, some small changes within the morphology of CS and Col fibers were observed on their surface, while PLA fibers remained unchanged (Figure 5a). Moreover, the mass loss evaluation presented in Figure 5b shows a similar degradation trend of monolith and core-shell fibers, with a mass loss of 5.40% ± 0.28% for PLA and 6.41% ± 0.91% for CS fibers, and the smallest difference in mass loss rate observed for these two types of mats after 10 days, while the degradation of Col fibers was much more pronounced with 18.13% ± 3.13% loss after 14 days.

### 3.5 Biocompatibility

**FIGURE 6:**
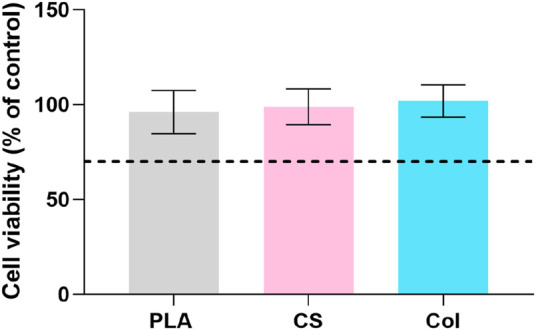
Biocompatibility of the scaffolds. The dashed line indicates the 70% cytotoxicity threshold according to ISO10993-5.

Figure 6 presents the viability of L929 murine fibroblasts treated with extract obtained from PLA, CS, and Col fibrous constructs for 24 h. All materials were found to be biocompatible with viability of 96%, 99% and 102%, for PLA, CS, and Col scaffolds, respectively.

### 3.6 DNA content calculated from MSCs differentiation culture

**FIGURE 7:**
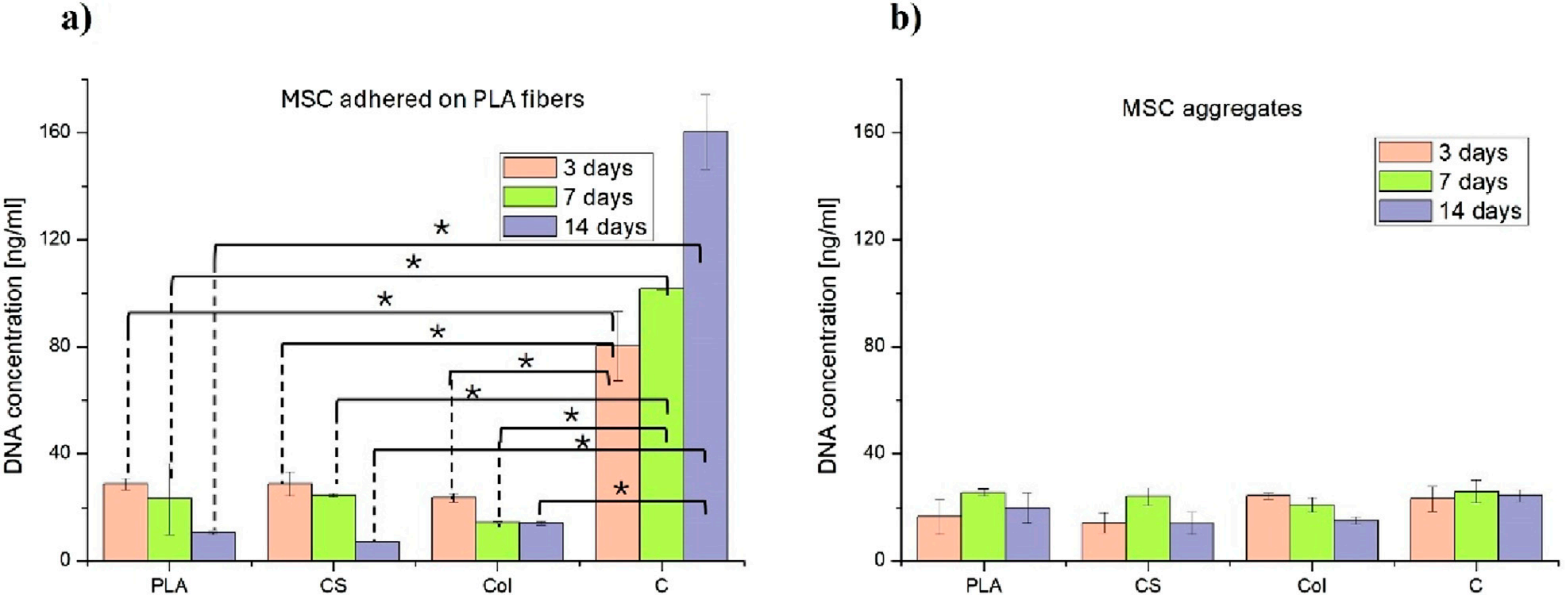
DNA concentration [ng/mL] for: **(a)** cells adhered to PLA fabrics, and **(b)** cell aggregates floating in the medium above the corresponding PLA fabrics. Statistical significances were indicated for the values obtained for the corresponding culture timepoints. Statistical analysis was performed by one-way analysis of variance (ANOVA) and *post hoc* Tukey’s test * indicates significance when p ≤ 0.05 (n = 4).

**FIGURE 8:**
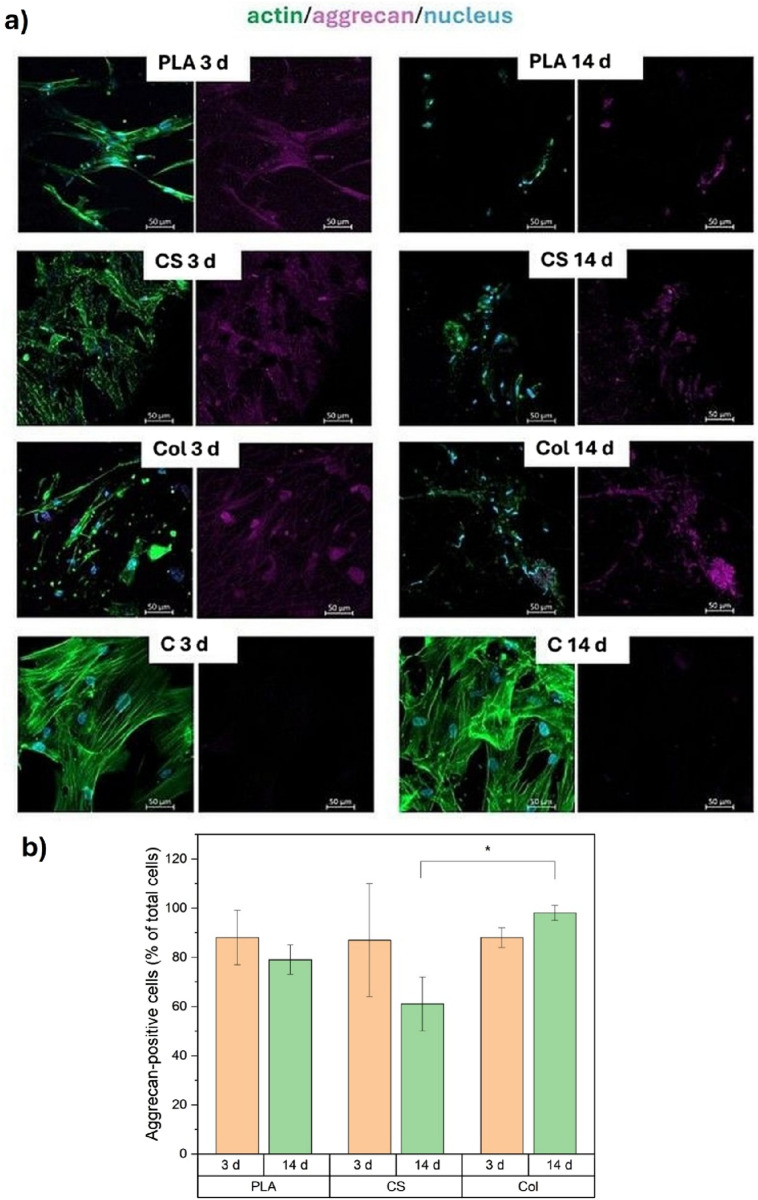
**(a)** confocal microscopy of MSCs after 3 and 14 days of chondrogenic differentiation on the surface of the tested materials. Scale bar: 50 µm (magnification ×200). Legend: green–actin fibers (cytoskeleton); purple–aggrecan; blue–cell nuclei, **(b)** Quantification of aggrecan-positive cells. The bar graph shows the mean percentage of aggrecan-positive cells relative to the total number of nuclei, averaged across multiple confocal images for each scaffold type (PLA, CS, Col) at two culture time points (3 and 14 days). Data are presented as mean ± standard deviation (SD) from at least three independent images per sample variant. Statistical analysis was performed by one-way analysis of variance (ANOVA) and *post hoc* Tukey’s test, * indicates significance when p ≤ 0.05.

The concentration of DNA in cells that had adhered to fibrous mats and in the corresponding cell aggregates was found to be below 40 ng/mL, representing a reduction of approximately 2.75-fold compared to the glass control (Figure 7). This indicates that both the adhesion and proliferation of cells were low on PLA-based fabrics of all types, which resulted in a minimal overall number of differentiating MSCs being detected in the presence of the tested PLA materials. This finding was corroborated by confocal microscopy (see Figure 8). Moreover, from the analysis of statistical significances, it can be seen that DNA content from the cells adhered to C (glass) was significantly different from other materials for all timepoints. For aggregates, there were no significant differences.

### 3.7 MSCs morphology

Immunostaining of actin and nuclei was employed to visualize cell morphology and aggrecan presence in differentiating mesenchymal stem cells. Representative confocal images are presented in Figure 8a. Following a 3-day culture period, the cells exhibited a flattened morphology on PLA and CS materials, in contrast to the limited cell flattening observed on the Col variant. Aggrecan was present in all tested materials apart from the control, which is to be expected given that aggrecan is a chondrogenic differentiation marker. After 14 days of *in vitro* culture, cell adhesion was significantly reduced on all PLA fabrics. However, aggrecan was still detected, especially on the Col variant. In control cells, adhesion was observed to occur properly. The cell content was higher than that observed on the tested fabrics, but aggrecan was not detected.

As quantified from confocal images, the fraction of aggrecan-positive cells is summarized in Figure 8b. Aggrecan-positive cells adhered to Col showed a significant increase from day 3 to day 14, while the fraction of said cells on PLA and CS did not change significantly.

### 3.8 Glycosaminoglycans (GAG) content

**FIGURE 9:**
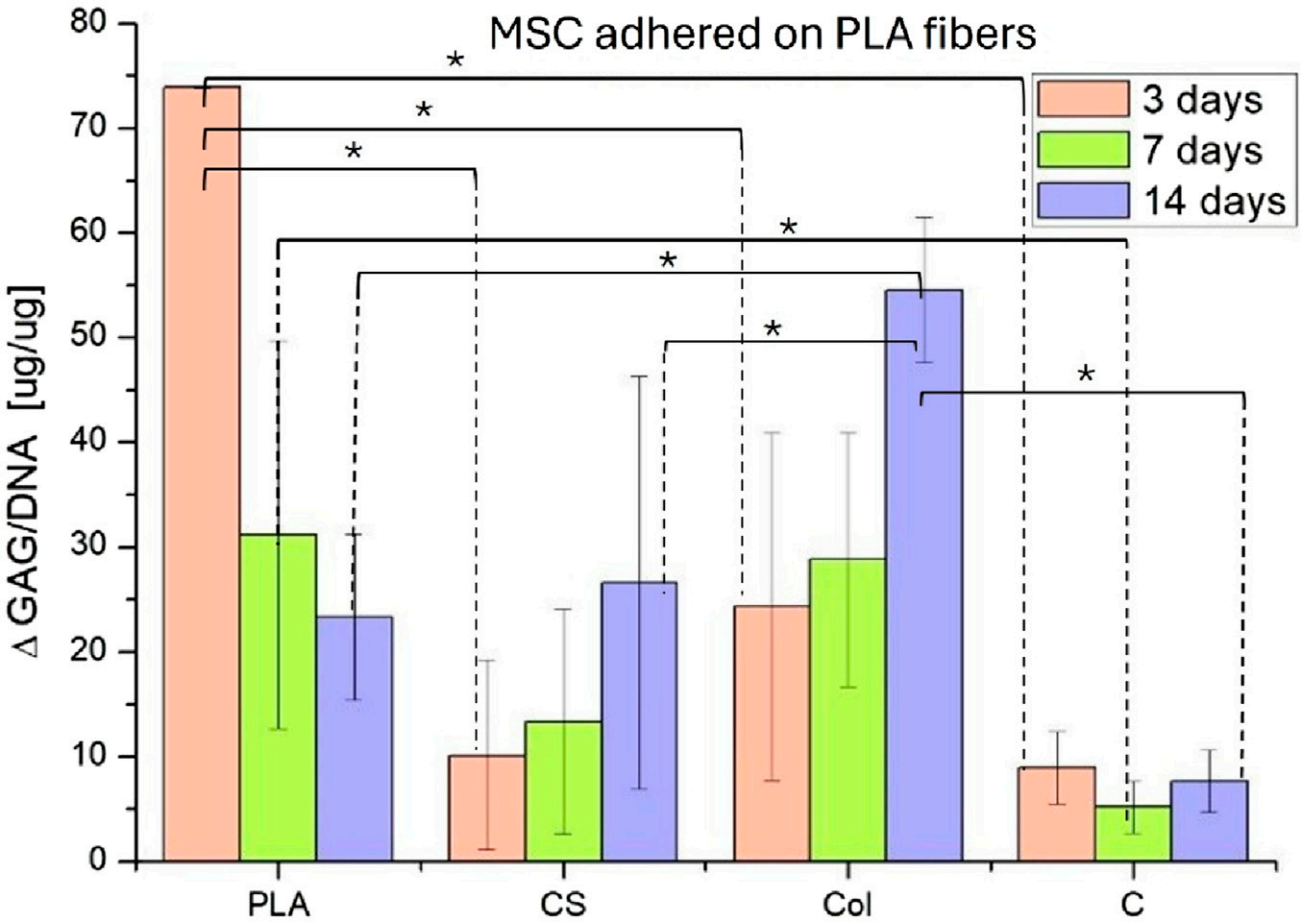
ΔGAG/DNA ratio [µg/µg] on all tested and control materials. Statistical significances were indicated for the values obtained for the corresponding culture timepoints. Statistical analysis was performed by one-way analysis of variance (ANOVA), and *post hoc* Tukey’s test* indicates significance when p ≤ 0.05 (n = 4).

The increase of GAG content presented as ΔGAG/DNA (increase of GAG amount per DNA mass) was found to be significantly greater in all tested materials in comparison to the control (Figure 9). In the case of PLA monolith fibers, the increase of ΔGAG/DNA exhibited a decline throughout the culture. In the case of CS and Col materials, ΔGAG/DNA increased for the culture period, while the DNA content decreased (Figure 7). The highest GAG content was produced by MSCs on Col mats after 14 days of culture. In contrast, ΔGAG/DNA on the control remained relatively low throughout the culture, despite the high DNA content increase. The analysis of statistical significance revealed that for 3 days of culture, GAG content on PLA significantly differed from other variants, for 7 days–the only significant difference was between PLA and C, and for 14 days–there were significant differences between Col and every other variant.

## 4 Discussion

Biomaterial-based platforms are gaining increasing interest in cartilage tissue engineering, offering a potential environment for supporting the chondrogenic differentiation of mesenchymal stem cells. Among these, electrospun mats - EBPB emerge as a promising approach due to their fibrous architecture, which resembles the native ECM and provides structural support for cell attachment and matrix deposition. However, their full potential as functional platforms for chondrogenesis still requires optimization in terms of composition, mechanical properties, and bioactive modifications to effectively guide cell behaviour and promote long-term cartilage regeneration.

The objective of this study was to assess whether the incorporation of PRP and collagen I into PLA electrospun fibers can facilitate the chondrogenic differentiation of MSCs. Three different types of PLA-based fibrous mats have been fabricated using the electrospinning method: monolith pure polymeric PLA, blended PLA-collagen, and PRP-PLA core-shell fibers.

Electrospinning allows for the fabrication of fibers with bioactive agents integrated throughout their entire volume, ensuring a homogeneous distribution within the material. In conventional electrospinning, these agents are blended directly into the polymer solution, resulting in fibers where the bioactive components are embedded within the fiber matrix. However, in coaxial electrospinning, a specialized setup with two concentric nozzles is used to produce core-shell fibers, where the core can encapsulate fragile bioactive ingredients while the outer shell provides a protective barrier (Kijeńska and Święszkowski, 2017). This approach helps safeguard sensitive molecules, such as proteins or growth factors, from degradation while enabling their controlled and sustained release, making it a promising strategy for biomedical applications (Xue et al., 2018; Maleki et al., 2020; Maleki et al., 2022). In our study, we utilized the advantages of both techniques to fabricate fibers enriched with bioactive natural compounds, ensuring their effective incorporation while maintaining bioactivity. Standard electrospinning has been utilized to produce pure PLA and blended PLA-collagen fibers, while coaxial electrospinning has been employed to produce PLA shell fibers with encapsulated PRP, to allow their preservation and prolonged growth factor release into MSCs. Fabricated PLA and Col fibers possessed similar diameters of 1.43 ± 0.59 µm for PLA and 1.50 ± 0.54 µm for Col, respectively, while the utilization of a coaxial setup resulted in decreased diameters of the obtained CS fibers to 0.73 ± 0.46 µm. This phenomenon can be attributed to the altered viscoelastic and electrohydrodynamic properties inherent in coaxial electrospinning. The introduction of a core-shell structure modifies charge distribution and jet dynamics, leading to increased stretching and thinning of the fibers. Additionally, the presence of a lower-viscosity core solution can facilitate greater elongation of the outer shell during electrospinning, further contributing to diameter reduction. Similar effects have been observed in previous studies on coaxial electrospinning, where modifications in solution properties and processing conditions influenced fiber morphology (Becker et al., 2015; Lauricella et al., 2017). The distribution method of the natural compounds within the fibers also determines other properties of the resultant mats, such as wettability. The encapsulation of PRP within the PLA shell did not change the hydrophilicity of the material, so PLA and CS samples maintained a similarly hydrophobic surface with WCA of 120.12° ± 0.77° and 117.64° ± 3.20°, respectively. In contrast, in Col mats, where the protein was incorporated into the fibers during the fabrication process, its presence significantly reduced the water contact angle to 80.04° ± 9.44°, resulting in a hydrophilic surface. This increase in hydrophilicity is primarily due to the inherent water-attracting nature of proteins, which possess numerous hydrophilic functional groups such as amino groups. These groups facilitate hydrogen bonding with water molecules, thereby reducing the water contact angle and rendering the surface more hydrophilic (Song and Mano, 2013). The presence of the amino groups of amide I and amide II on the surface of the Col fibrous mats has been confirmed by the FTIR evaluation, and the characteristic bands were present at 1,653 cm^−1^ and 1,541 cm^−1^, respectively. Moreover, the structure of the produced fibers influenced the tensile properties in terms of their ductility. The incorporation of the collagen and PRP disrupted the uniform polymer structure and, as a result, decreased the tensile stress of the fibrous mats. In the case of the PLA fibers, the absence of the natural compound allowed for uniform chain alignment and, thus, their greater flexibility.

Collagen I is a major natural polymer occurring in the ECM of cartilage tissue and is relevant for its normal structural integrity. PRP, on the other hand, contains a plethora of bioactive components, including numerous growth factors (e.g., epidermal growth factor (EGF), insulin-like growth factor 1 (IGF-1), hepatocyte growth factor (HGF), transforming growth factor β (TGF-β)), adhesion proteins, chemokines, and other biological mediators (Diaz-Gomez et al., 2014; Pötter et al., 2021). Both have been proven to support MSCs chondrogenic differentiation, as described in the Introduction section. In our study, there was a significant mass loss of Col mat. It can be linked to collagen release and dissolution in culture medium. It was demonstrated that said mass loss did not restrict MSCs chondrogenic differentiation, as it was maintained on Col mat for 14 days, with the best yield among all the tested materials. Additionally, Col fibrous mats started to become brittle after 10 days of incubation in PBS. Although the loosening of the fibrous structure was visible on the SEM image, the integrity thereof was intact.

As for PRP, it has also been demonstrated that it has a beneficial effect on the chondrogenic differentiation of MSC cells, even in the absence of other differentiation factors such as hypoxia (Wang et al., 2019), and also when PRP was incorporated into a polymeric fibrous mat (Diaz-Gomez et al., 2014). In the present study, the PRP protein release profile indicated the controlled secretion of proteins from the fiber core, since it was released for more than 24 h. However, the plateau phase was reached on day 3, which is approximately 10 times faster than the PRP release reported in other studies (Chen et al., 2023). Despite this, as is described below, it was possible to maintain chondrogenic lineage for 14 days of MSCs culture. Additionally, the biodegradation studies showed a similar trend of degradation for both PLA and CS fibers, with the biggest difference in mass loss between the two materials after 10 days, which might be associated with the released PRP from the core of the core-shell structure.

Despite a short period of PRP release, it was still possible to maintain and even enhance chondrogenic lineage for 14 days of MSC culture in comparison to PLA mats not releasing any active agents. The result is satisfactory when considered in relation to the results reported in the relevant literature. Bolandi et al. (2021) applied an injectable hydrogel, based on alginate/polyvinyl alcohol incorporating platelet-rich plasma (PRP)-encapsulated alginate sulfate microbeads, as a localized sustained release system of growth factors for the articular cartilage regeneration (Bolandi et al., 2021). Similar to our study, the total released protein amount was quantified to show PRP release (with Bradford assay), and no quantification of specific growth factors was applied. The authors detected sustained release of PRP for 14 days, but what is important, PRP was encapsulated in two types of microbeads incorporated in a hydrogel matrix, whereas we did not apply any microbead encapsulation. Moreover, Shen et al. (2015) reported that MSCs cultured on a PRP gel scaffold increased expression of type II collagen, SOX9, and aggrecan as early as 7 days, with Safranin O staining and immunohistochemistry indicating glycosaminoglycan and collagen formation (Shen et al., 2015). Next, mentioned high SOX9 expression at 14 days in a crosslinked hyaluronic acid/chondroitin sulfate/carboxymethyl chitosan hydrogel infused with PRP (KhaliliJafarabad et al., 2022). These findings indicate that, when appropriately formulated, PRP-releasing biomaterials can promote early chondrogenic markers in MSCs within a 2-week interval. Of course, there are works mentioning longer *in vitro* cultures, as well as short-, mid-, and long-term *in vivo* studies. In our opinion, it depends on the stage of the research. In our preliminary study, we demonstrated that the chondrogenic differentiation enhancement can be shown even in a relatively short *in vitro* culture, which we consider another strength of the research.

While designing studies for the development of a platform with specific properties, the parameters of the biological evaluation should be carefully constructed. In our study, a DMEM-based differentiation medium with the addition of 10 ng/mL TGF-β3 was applied for MSCs culture of PLA fibers, whereas for MSCs culture on glass controls, a regular DMEM medium was used. The glass with non-differentiation medium was utilized to evaluate GAG/DNA and aggrecan basal levels, as well as MSCs morphology in the non-differentiation environment. In this study, the material control comprised PLA mats with differentiation medium, indicating that the MSCs were cultivated on a standard polymeric fabric in well-established differentiation conditions. Finally, the objective was to emphasise the superior effect of MSCs differentiation over typical PLA fabric in typical differentiation conditions, as evidenced by Col (PLA blended with collagen) and CS (core-shell fabric releasing PRP). The differentiation medium was applied to the Col and CS cultures, thereby providing a single change (material type) at the time. Thanks to such an experimental design, interesting outcomes could be measured. Performed comparison of the culturing of MSCs on the fibrous mats of different types led to the observation that the concentration of DNA in cells that had adhered to PLA-based fibrous mats of all types and in the corresponding cell aggregates was found to be below 40 ng/mL, representing a reduction of approximately 2.75-fold compared to the glass control. This indicates that the proliferation of cells was low on PLA fibers. In the case of Col, the DNA content decline was observed until day 7 of culture and then stopped. These results are consistent with what is going on during the chondrogenic lineage of MSCs. The chondrogenic differentiation of MSCs includes five main stages: condensation, differentiation, proliferation, hypertrophy, and angiogenesis. First, in the presence of certain paracrine factors, MSCs produce an extracellular matrix containing hyaluronan, collagen type I, and collagen type II, and then undergo increased condensation through cell-ECM and cell-cell interactions. Second, MSCs differentiate into chondrocytes, and ECM components, like aggrecan, are secreted. Third, differentiated chondrocytes proliferate rapidly and continue to produce ECM, including aggrecan. Fourth, mature chondrocytes take on a hypertrophic phenotype and begin to express collagen type X and alkaline phosphatase. Fifth, hypertrophic chondrocytes are replaced with blood vessels after cell death (Zha et al., 2021; Goldring et al., 2006). According to the given description, intense proliferation occurs in stage 3^rd^, after early chondrogenic differentiation. Other sources claim that the proliferation phase has to begin before chondrogenesis to increase the local cell density, thus inducing chondrogenic differentiation (Gao et al., 2017). However, in the present study, we initially provided MSCs condensation with the micromass culture technique. Consequently, the absence of low proliferation, as indicated by plateau DNA content, does not preclude the occurrence of a proper differentiation process. The established differentiation process is further confirmed by the production of GAG and aggrecan (discussed below). The statement that the secretion of GAGs is a marker of the presence of mature chondrocytes is supported by other research (Zhong et al., 2015). Moreover, the high proliferation rate observed in control and not observed in the electrospun fibrous mats indicates that in the absence of any differentiation-inducing factors, MSCs only proliferated as they were under MSCs expansion culture conditions.

The decision to set the differentiation time to 14 days was based on several considerations. Firstly, numerous studies have demonstrated that key indicators of chondrogenic differentiation—such as collagen II and aggrecan expression—can be reliably detected within the first 14 days of culture in 3D aggregate or scaffold-based systems. For example, Lei et al. reported that COL2A1 mRNA levels and matrix deposition became significantly elevated by day 14 and showed only modest further increase at later timepoints. Notably, COL10A1 (a marker of hypertrophy) also became detectable by day 14 and differentiated between conditions, indicating that early phenotypic divergence is already observable within this timeframe (Lei et al., 2016). This is further supported by studies in other models, such as canine MSCs spheroid cultures, where strong Safranin O staining and robust COL2 expression—but no detectable COL10—were observed at day 14 under chondrogenic induction. These findings were accompanied by clear activation of SOX9 and ACAN expression at that point (Legendre et al., 2017; Endo et al., 2019). Secondly, our study focused specifically on assessing early chondrogenic commitment and the influence of scaffold composition on lineage direction. Collagen II and aggrecan are reliably upregulated by day 14 (Lei et al., 2016). Thus, our chosen timepoint was appropriate for detecting biologically meaningful differences in scaffold-induced differentiation. Lastly, extending culture beyond 14 days on PLA-based scaffolds without repeated cytokine supplementation or medium refreshment can introduce variability related to nutrient depletion or scaffold degradation, rather than reflecting material performance. To ensure consistent and controlled conditions across groups, we selected day 14 as a standardized, literature-supported endpoint for this study.

**FIGURE 10:**
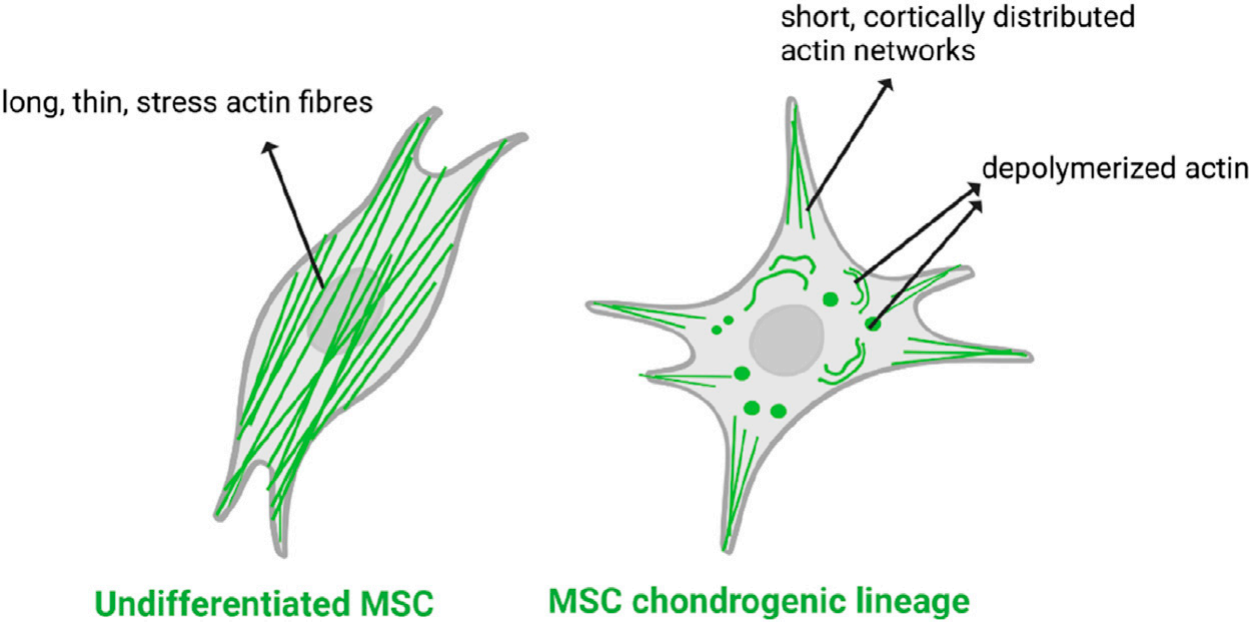
Schematic representation of actin filaments remodelling in undifferentiated MSCs and the chondrogenic lineage of MSCs.

Figure 8a presents the morphology of differentiated MSCs adhered to the materials’ surface, as well as the presence of aggrecan secreted by the cells. It can be seen that the actin fibers reorganized during the culture. After 3 days of culture, MSCs presented long, thin, parallel stress fibers. On day 14, the cells showed short, disorganized actin filaments with cortical localization (close to the cell membrane (Chalut and Paluch, 2016)). Such actin rearrangement has been proven to be an important marker of chondrogenic differentiation (Putra et al., 2023; Yang et al., 2020). It was observed that *in vitro*, MSCs differentiated towards a chondrogenic lineage, and by day 14 the cytoskeleton had altered, with a loss of stress fibers and an increase in cortical actin (Cheng et al., 2018). What is more, in the present study, actin filaments in MSCs on day 14 are not only shorter and disoriented but also manifest a point-like structure. It can be interpreted as the sign of actin depolymerization (Magnussen et al., 2021; Sen et al., 2024), which is also a typical phenomenon occurring in the chondrogenic differentiation process, according to the hypothesis that a decrease in cellular rigidity after the depolymerization of actin filaments favors adipogenesis or chondrogenesis of MSCs (Putra et al., 2023; Saidova and Vorobjev, 2020). Said actin remodelling is shown schematically in Figure 10 below. The synthesis of aggrecan was also confirmed with confocal imaging (Figure 8), although it seems that it was localized intracellularly rather than secreted. This is a common situation in in vitro MSCs chondrogenesis, and it was observed in, e.g., 2–3 weeks MSCs culture (Park et al., 2016) and in long-term chondrogenic micromass culture (Xu et al., 2016). It was discussed that applying any mechanical stimuli, such as static or dynamic compression, as well as more rigid polymeric scaffolds for MSCs lineage, could support aggrecan secretion (Le et al., 2020).

The image-based quantification of aggrecan-positive cells (Figure 8b) supported our qualitative observations. Col mats not only better sustained, but might have even enhanced aggrecan expression over 2 weeks, while PLA and CS did not show a significant increase in this expression over time. This suggests that the cell-matrix environment provided by collagen was more conducive to maintaining a chondrogenic phenotype under our culture conditions. Although this assay measured the proportion of aggrecan-positive cells rather than the total output of the matrix, the observed trend was consistent with other results.

Prior studies of MSCs-derived chondrocytes have documented extensive intracellular retention of aggrecan within the endoplasmic reticulum (ER) during early differentiation stages, visualized clearly via confocal immunofluorescence—without necessarily correlating these observations to secreted ECM at later time points. For instance, chondrocytes derived from both BM-MSCs and iPSCs were shown to accumulate large amounts of aggrecan intracellularly during early differentiation, as observed by confocal microscopy in cultures up to day 35, with mature secretion occurring later (Krawetz et al., 2022; Xu et al., 2016). A similar pattern has been seen in other MSCs-derived chondrocyte systems where intracellular aggrecan localized in the ER was interpreted as evidence of active matrix synthesis in early chondrogenesis. Given our study focuses on the early scaffold-induced chondrogenic commitment and intracellular protein synthesis dynamics (rather than matured ECM assembly), we chose confocal visualization of intracellular aggrecan as a valid and sufficient surrogate indicator of biosynthetic activity. These findings align with published models that also relied primarily on intracellular immunolocalization to support functional phenotyping.

Some interesting outcomes were obtained from ΔGAG/DNA analysis. For PLA, ΔGAG/DNA was the highest on day 3 of culture and then decreased, whereas for CS and Col, it increased, reaching the peaks at the 14-day timepoint. For pure PLA, the GAG production was the most intense during the first 3 days of culture, when the chondrogenic differentiation was maintained with the DMEM differentiation medium with the addition of TGF-β3. However, after that, the GAG secretion decreased, in contrast to the GAG production measured on CS and Col variants. The explanation is that the gradual release of PRP and Col presence facilitated and sustained chondrogenic lineage commitment of MSCs for a longer period than just the utilization of the differentiation medium. The highest ΔGAG/DNA on 14 days of culture was observed for the material containing collagen I, indicating that this bioactive component may be a more suitable stimulus for differentiation than PRP. Also, for 14 days of culture, the only significant difference in GAG content was indicated between Col and every other variant. At first, the GAG content was significantly higher on PLA, but after 14 days the effect of collagen I in Col surpassed the impact of PLA mat. The normalized GAG levels obtained are comparable to the amounts measured in other studies. For MSCs from different donor organisms cultured in spheroids for 21 days in the chondrogenic lineage, ΔGAG/DNA reaching 40 μg/μg was considered high (Lam et al., 2018), whereas in the present study, it achieved 26.6 μg/μg for CS and 54.5 μg/μg for Col after 14 days. In another study on gelatin-based scaffolds for MSCs chondrogenesis, the highest amount of secreted GAG after 14 days was 0.25 ng/cell (Menezes and Arinzeh, 2020). Assuming that in our study, MSCs was not proliferating and in the culture course, there were 10^5^ cells per material, after 14 days, 0.266 ng GAG/cell for CS and 0.545 ng GAG/cell for Col. Thus, in the light of similar research, the GAG content obtained in this study is very similar for CS and twice higher for Col material.

The current state of the art confirms that collagen and PRP can be used to induce stem cell differentiation. PRP has been demonstrated to induce GAG production by promoting chondrogenic differentiation, thereby circumventing the hypertrophy side effect observed in chondrocytes. The hypertrophy phenomenon has the potential to result in the undesirable ossification of cartilage tissue, which is an unfavourable outcome in the context of cartilage treatment (Hesari et al., 2020). This highlights the superiority of PRP over the TGF-β3 growth factor, which is frequently employed as a chondrogenic differentiation trigger. The utilization of PRP in stem cell therapy for cartilage defects has resulted in the desired outcomes, including the expeditious replenishment of GAG deficiencies (Le et al., 2020). As for collagen I, given that it is a component of the extracellular matrix (ECM), it facilitates cell adhesion at the surface, thereby initiating and supporting chondrogenic differentiation (Xia et al., 2018). Furthermore, it has been demonstrated that this compound is capable of interacting with integrin, which additionally promotes differentiation. The presence of collagen I resulted in significantly elevated differentiation levels of human MSCs in a 30-day *in vitro* culture, exceeding those observed with fibronectin and fibrinogen (Linsley et al., 2013). Similar findings were reported by Lanfer et al., whereby the GAG content in MSCs culture was elevated in media with aligned and non-aligned Col I, suggesting that chondrogenic differentiation was enhanced in the tested media (Lanfer et al., 2009). The objective of recent research is to incorporate collagen into the scaffolds used for cartilage tissue regeneration. Calabrese et al. reported an increase in chondrogenic differentiation of human adipocytic stem cells on a scaffold created from horse collagen I, which has the potential to facilitate the generation of new cartilage tissue (Calabrese et al., 2017).

## 5 Conclusion

In the presented study, we successfully fabricated PLA-based fibrous mats enriched with bioactive compounds, capable of releasing PRP. Moreover, the incorporation of PRP and collagen I into the PLA fibrous mats supported MSCs differentiation for 14 days, achieving levels comparable to or even exceeding those reported in previous studies. Additionally, GAG secretion in Col samples was twice as high after 14 days compared to similar fibrous mat-based approaches. The presence of aggrecan, a key chondrogenic differentiation marker, was more pronounced in CS and Col samples than in pure polymeric PLA, further confirming enhanced chondrogenesis. Furthermore, cytoskeletal changes, including actin depolymerization, were observed—an important indicator of chondrogenic differentiation. These findings highlight the potential of PRP or collagen-enriched PLA fibrous mats as promising platforms for cartilage tissue engineering.

## Data Availability

The original contributions presented in the study are included in the article/Supplementary Material, further inquiries can be directed to the corresponding authors.
