# Correction: Electrospun bioactive polymer biomaterials enriched with collagen and platelet-rich plasma as a platform for *in vitro* chondrogenic differentiation of human mesenchymal stem cells

**DOI:** 10.3389/fbioe.2025.1770030

**Published:** 2026-01-19

**Authors:** Paulina Trzaskowska, Ewa Rybak, Kamil Kopeć, Tomasz Ciach, Piotr Wieciński, Wojciech Święszkowski, Ewa Kijeńska-Gawrońska

**Affiliations:** 1 Centre for Advanced Materials and Technologies CEZAMAT, Warsaw University of Technology, Warsaw, Poland; 2 Faculty of Chemical and Process Engineering, Warsaw University of Technology, Warsaw, Poland; 3 Faculty of Chemistry, Warsaw University of Technology, Warsaw, Poland; 4 Faculty of Materials Science and Engineering, Warsaw University of Technology, Warsaw, Poland

**Keywords:** electrospinning, cartilage tissue engineering, mesenchymal stem cells, chondrogenic differentiation, platelet-rich plasma

The reference for “Cipolletta et al., 2016” was erroneously written as “Cipolletta, F., Lauricella, M., National, I., Pontrelli, G., National, I., and Pisignano, D. (2016). Effects of orthogonal rotating electric fields on electrospinning process. *Phys. Fluids (1994).* 29, 082003. doi:10.1063/1.4997086”. It should be “Lauricella, M., Cipolletta, F., Pontrelli, G., Pisignano, D., and Succi, S. (2017). Effects of orthogonal rotating electric fields on electrospinning process. Phys. Fluids 29, 082003. doi:10.1063/1.4997086”.

The reference for “Vorobjev and Saidova, 2019” was erroneously written as “Vorobjev, I. A., and Saidova, A. A. (2019). Lineage commitment, signaling pathways, and the cytoskeleton systems in mesenchymal. *stem cells* 7, 1–44. doi:10.1089/ten.TEB.2019.0250”. It should be “Saidova, A. A., and Vorobjev, I. A. (2020). Lineage commitment, signaling pathways, and the cytoskeleton systems in mesenchymal stem cells. Tissue Eng. Part B Rev. 26(1), 13–25. doi:10.1089/ten.teb.2019.0250”.

The reference for “Silva et al., 2010” was erroneously written as “Silva, M. L. A., Martins, A., Costa, P., Faria, S., Gomes, M., Reis, R. L., et al. (2010). Cartilage tissue engineering using electrospun PCL Nanofiber meshes and MSCs. *Biomacromolecules* 11, 3228–3236. doi:10.1021/bm100476r”. It should be “Alves da Silva, M. L., Martins, A., Costa-Pinto, A.R., Costa, P., Faria, S., Gomes, M, Reis RL, et al. (2010). Cartilage tissue engineering using electrospun PCL nanofiber meshes and MSCs. Biomacromolecules 11, 3228–3236. doi: 10.1021/bm100476r”.

The reference for “Kijenska and Eva, 2017)” was erroneously written as “Kijenska, W., and Ewa, S. (2017). “General requirements of electrospun materials for tissue engineering: setups and strategy for successful electrospinning in laboratory and industry,” in Electrospun mater. Tissue Eng. Biomed. Editors E. Uyar, and K. Tamer (Appl.), Woodhead Publishing. 43–56. doi:10.1016/B978-0-08-101022-8.00002-8”. It should be “Kijeńska, E., and Święszkowski, W. (2017). 2 - General requirements of electrospun materials for tissue engineering: setups and strategy for successful electrospinning in laboratory and industry. In: Electrospun Materials for Tissue Engineering and Biomedical Applications. Eds. E. Uyar and E. Kny. Cambridge: Woodhead Publishing, 43–56. doi:10.1016/B978-0-08-101022-8.00002-8”.

The reference for “Kijenska-Gawr et al., 2019” was erroneously written as “Kijenska-Gawronska, W., Bolek, T., Bil, M., and Swieszkowski, W. (2019). Alignment and bioactive molecule enrichment of bio-composite scaffolds towards peripheral nerve tissue engineering. J. Mat. Chem. B 7, 4509–4519. doi:10.1039/c9tb00367c”. It should be “Kijeńska-Gawrońska, E., Bolek, T., Bil, M., and Święszkowski, W. (2019). Alignment and bioactive molecule enrichment of bio-composite scaffolds towards peripheral nerve tissue engineering. J. Mater. Chem. B 7, 4509–4519. doi:10.1039/C9TB00367C”.

The reference for “ISO 10993-5, 2015” was erroneously written as “ISO 10993-5 (2015). Biological evaluation of medical devices — part 5: tests for *in vitro* cytotoxicity. *Int. Organ. Stand.* 10406–1 (20), 3–6”. It should be “International Organization for Standardization. (2009). ISO 10993-5:2009 – Biological evaluation of medical devices – Part 5: Tests for *in vitro* cytotoxicity. Geneva: ISO”.

The reference for “International Organization for Standardization, 2004” was erroneously written as “International Organization for Standardization (2004). ISO 10993 Biological evaluation of medical devices — part 12: sample preparation and reference materials. Iso 10993-12 3, 1–20. Available online at: http://down.40777.cn/stardard/11/ISO10993.12-2004Biologicalevaluationofmedicaldevices.pdf”. It should be “International Organization for Standardization. (2012). ISO 10993-12:2012 – Biological evaluation of medical devices – Part 12: Sample preparation and reference materials. Geneva: ISO”.

The reference for “Cheng et al., 2017” was erroneously written as “Cheng, A., Cain, S. A., Tian, P., Baldwin, A. K., Uppanan, P., Kielty, C. M., et al. (2017). Recombinant extracellular matrix protein fragments support Human embryonic stem cell chondrogenesis. Tissue Eng. Part A 24, 968–978. doi:10.1089/ten.TEA.2017.0285”. It should be “Cheng, A., Cain, S. A., Tian, P., Baldwin, A. K., Uppanan, P., Kielty, C. M., et al. (2018). Recombinant extracellular matrix protein fragments support human embryonic stem cell chondrogenesis. Tissue Eng. Part A 24, 968–978. doi:10.1089/ten.TEA.2017.0285”.

The reference for “Ding et al., 2020” was erroneously written as “Ding, H., Cheng, Y., Niu, X., and Hu, Y. (2020). Application of electrospun nanofibers in bone, cartilage and osteochondral tissue engineering. J. Biomater. Sci. Polym. Ed. 32, 536–561. doi:10.1080/09205063.2020.1849922”. It should be “Ding, H., Cheng, Y., Niu, X., and Hu, Y. (2021). Application of electrospun nanofibers in bone, cartilage and osteochondral tissue engineering. J. Biomater. Sci. Polym. Ed. 32, 536–561. doi:10.1080/09205063.2020.1849922”.

The reference for “Jiang et al., 2019” was erroneously written as “Jiang, J., Tongmeng, H., Shujun, H., Zheng, L., and Loh, X. J. (2019). Biomimetic Poly(Poly(E-caprolactone)- polytetrahydrofuran urethane) based nanofibers enhanced chondrogenic differentiation and cartilage regeneration, J. Biomed. Nanotechnol. 1005–1017. doi:10.1166/jbn.2019.2748”. It should be “Jiang, T., Heng, S., Huang, X., Zheng, L., Kai, D., Loh, X. J., et al. (2019). Biomimetic poly(ε-caprolactone)-polytetrahydrofuran urethane-based nanofibers enhance chondrogenic differentiation and cartilage regeneration. J. Biomed. Nanotechnol. 15, 1005–1017. doi:10.1166/jbn.2019.2748”.

The reference for “Lam et al., 2018” was erroneously written as “Lam, S., Johnny, B., Ian, M., Ross, B., Steven, P., and Raj, K. (2018). Functional profiling of chondrogenically induced multipotent stromal cell aggregates reveals transcriptomic and emergent morphological phenotypes predictive of differentiation capacity. Stem Cells Transl. Med., 1–12. doi:10.1002/sctm.18-0065”. It should be “Lam, J., Bellayr, I. H., Marklein, R. A., Bauer, S. R., Puri, R. K., and Sung, K. E. (2018). Functional profiling of chondrogenically induced multipotent stromal cell aggregates reveals transcriptomic and emergent morphological phenotypes predictive of differentiation capacity. Stem Cells Transl. Med. 7, 664–675. doi:10.1002/sctm.18-0065”.

The reference for “Lei et al., 2016” was erroneously written as “Lei, J., Jennifer, T., and Elda, T. (2016). Cell number and chondrogenesis in human mesenchymal stem cell aggregates is affected by the sulfation level of heparin used as a cell coating. J. Biomed. Mater Res. A 5, 1–8. doi:10.1002/jbm.a.35713.Cell”. It should be “Lei, J., Trevino, E., and Temenoff, J. S. (2016). Cell number and chondrogenesis in human mesenchymal stem cell aggregates is affected by the sulfation level of heparin used as a cell coating. J. Biomed. Mater. Res. A 104(7), 1817–1829. doi:10.1002/jbm.a.35713”.

The reference for “Menezes and Arinzeh, 2020” was erroneously written as “Menezes, T., and Roseline, A. (2020). Comparative Study of electrospun scaffolds containing native GAGs and a GAG mimetic for human mesenchymal. Stem Cell. Chondrogenes. doi:10.1007/s10439-020-02499-9”. It should be “Menezes, R., and Arinzeh, T. L. (2020). Comparative study of electrospun scaffolds containing native GAGs and a GAG mimetic for human mesenchymal stem cell chondrogenesis. Ann. Biomed. Eng. 48(7), 2040–2052. doi:10.1007/s10439-020-02499-9”.

The reference for “Song et al., 2013” was erroneously written as “Song, J., and Wenlong, M. (2013). Interactions between cells or proteins and surfaces exhibiting extreme wettabilities. Soft Matter 9, 2985. doi:10.1039/C3SM27739A”. It should be “Song, W., and Mano, J. F. (2013). Interactions between cells or proteins and surfaces exhibiting extreme wettabilities. Soft Matter 9(11), 2985–2999. doi:10.1039/C3SM27739A”.

The reference for “Xue et al., 2019” was erroneously written as “Xue, J., Wu, T., Dai, Y., Xia, Y., and States, U. (2018). Electrospinning and electrospun nanofibers: methods, materials, and applications. Chem. Rev. 119, 5298–5415. doi:10. 1021/acs.chemrev.8b00593”. It should be “Xue, J., Wu, T., Dai, Y., and Xia, Y. (2019). Electrospinning and electrospun nanofibers: methods, materials, and applications. Chem. Rev. 119, 5298–5415. doi:10.1021/acs.chemrev.8b00593”.

The original article has been updated.

