# Supplementary material for: Electrospun bioactive polymer biomaterials enriched with collagen and platelet-rich plasma as a platform for in vitro chondrogenic differentiation of human mesenchymal stem cells
Source: Front Bioeng Biotechnol. 2025 Dec 3;13:1629912. doi: 10.3389/fbioe.2025.1629912 (PMC12710238; doi:10.3389/fbioe.2025.1629912)
Supplement: Supplementary file 1 [file Supplementaryfile1.docx]

**Supporting information for “Electrospun bioactive polymer biomaterials enriched with collagen and platelet-rich plasma as a platform for *in vitro* chondrogenic differentiation of human mesenchymal stem cells”**

**Paulina Trzaskowska^1*^*,* Ewa Rybak^1^, Kamil Kopeć^1,2^, Tomasz Ciach^1,2,^ Piotr Wieciński^3^, Wojciech Święszkowski^4^, Ewa Kijeńska-Gawrońska^1*^**

^1^ Centre for Advanced Materials and Technologies CEZAMAT, Warsaw University of Technology, Poleczki 19, 02-822 Warsaw, Poland

^2^ Faculty of Chemical and Process Engineering, Warsaw University of Technology, Warynskiego 1, 00-645 Warsaw, Poland

^3^Faculty of Chemistry, Warsaw University of Technology, Noakowskiego 3, 00-664 Warsaw, Poland

^4^ Faculty of Materials Science and Engineering, Warsaw University of Technology, Wołoska 141, 02-507 Warsaw, Poland

* corresponding authors: [paulina.trzaskowska@pw.edu.pl](mailto:paulina.trzaskowska@pw.edu.pl), [ewa.kijeńska@pw.edu.pl](mailto:ewa.kijeńska@pw.edu.pl)


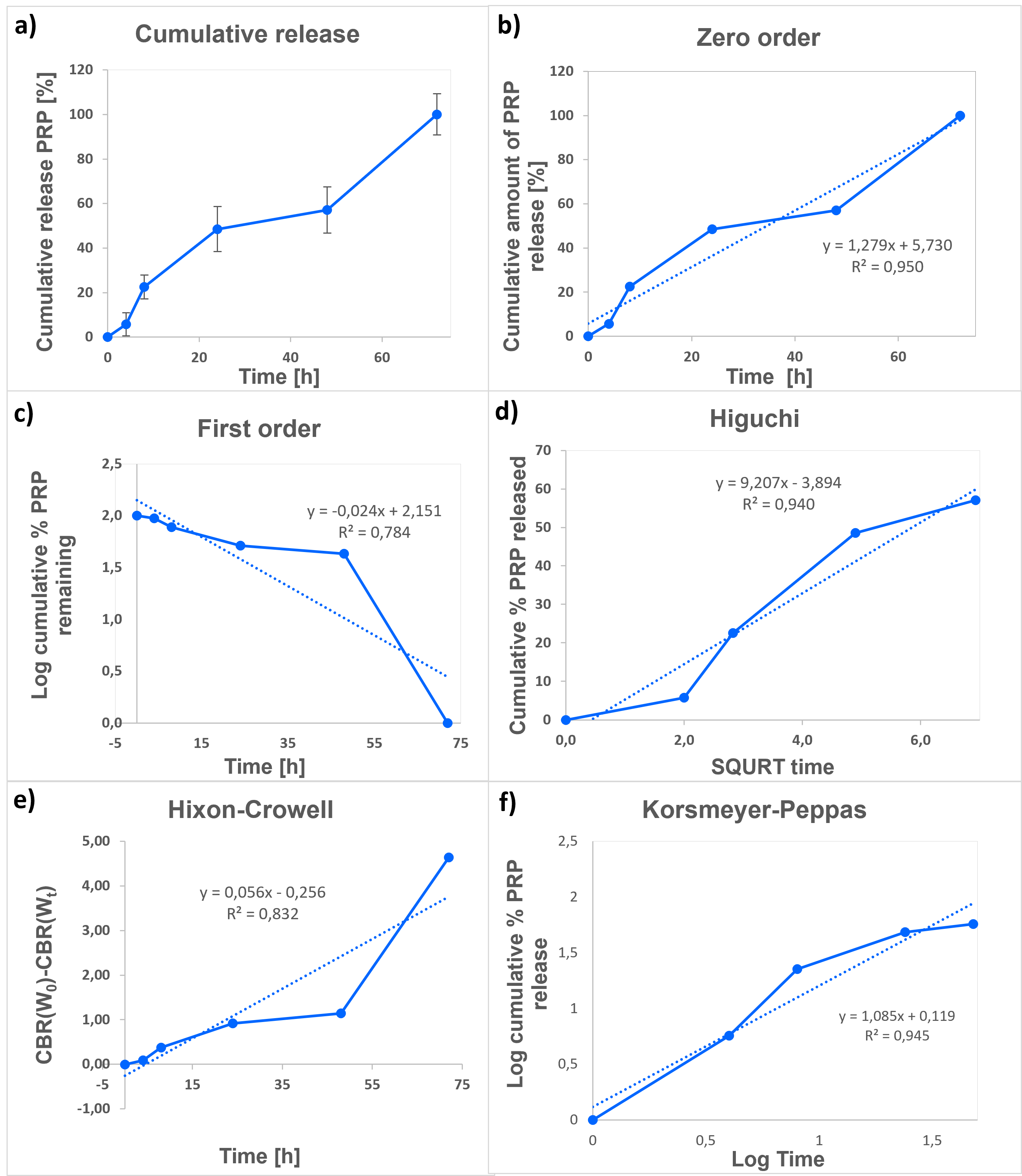
Figure S1. Cumulative release (a), Zero-order (b), First-order (c), Higuchi (d), Hixson-Crowell (e) and Korsmeyer-Peppas (f), kinetic models calculated for PRP release from fibrous CS constructs.
